# Rhabdomyolysis Induced by Levetiracetam: A Case Report in Kuwait

**DOI:** 10.1155/2024/1234738

**Published:** 2024-09-21

**Authors:** Moaz Qureshi, Prakash A. Abraham, Abdullah Al-Faras, Omar M. Bamasood, Kamal M. Matar

**Affiliations:** ^1^ Department of Internal Medicine Adan Hospital Ministry of Health, Kuwait City, Kuwait; ^2^ Department of Anesthesia Adan Hospital Ministry of Health, Kuwait City, Kuwait; ^3^ Department of Pharmacology and Therapeutics College of Pharmacy Kuwait University, Kuwait City, Kuwait

## Abstract

Epilepsy is a common disorder caused by a myriad of drugs, of that levetiracetam is being commonly used late because of its strong safety profile and efficacy. With the increasing usage of drugs, some rare side effects may sometimes appear that can escape the most stringent checks, possibly due to the rarity of their occurrence. Rhabdomyolysis is known to occur in some patients owing to a variety of causes, even leading to kidney injury. When a drug has a side effect that is not well recognized in the literature, especially when the side effect can mimic an adverse effect of an uncommon primary illness, identifying the causal factor can be doubly difficult. To date, only limited studies have been published suggesting rhabdomyolysis linked to levetiracetam use. We report the first case of levetiracetam-induced rhabdomyolysis in Kuwait.

## 1. Introduction

Levetiracetam is a commonly prescribed anti-seizure medication in clinical practice. The major side effects of this medication in adults include somnolence, headache, fatigue, accidental injury, infection, and dizziness [1]. Since receiving FDA approval in 1999, only limited case reports of levetiracetam-induced rhabdomyolysis have been published to date [1–9]. This side effect has not been described in long-term studies or randomized control trials, and only anecdotal evidence is available in the form of case reports, and more recently, a pharmacovigilance study [10]. As such, we report a case of a 30-year-old gentleman admitted with status epilepticus who developed levetiracetam-induced rhabdomyolysis.

## 2. Case Presentation

**Figure 1:**
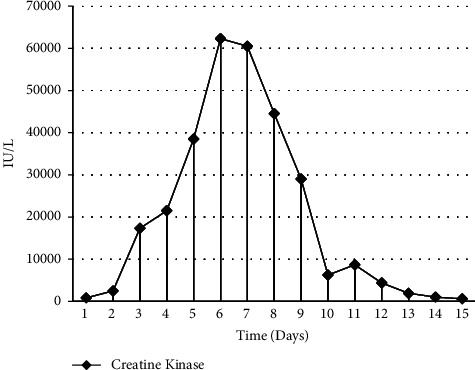
Serum creatinine kinase levels during hospitalization.

**Figure 2:**
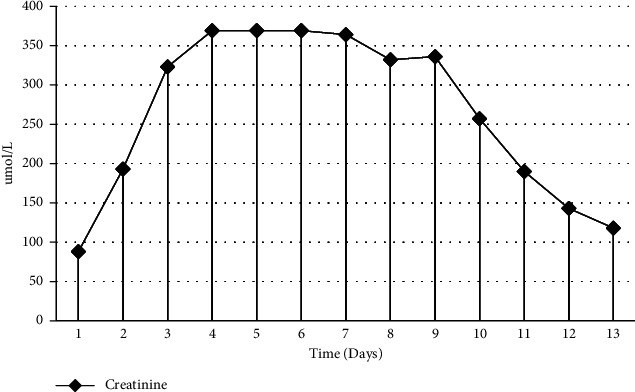
Serum creatinine profile during hospitalization.

The patient was a 30-year-old man who was brought to our hospital by emergency services after being found stuporous with frothing from the mouth at home. Upon presentation, there was no history available as the patient was drowsy and stuporous. The likely cause for this presentation was suspected to be postictal state secondary to seizure, and immediate care was initiated. The patient underwent emergency intubation upon arrival due to severe metabolic acidosis with a pH of 6.8, HCO_3_ of 5, lactate of 17, in conjunction with desaturation (saturation of 78%) due to decreased Glascow Coma Scale (4/15). He was then shifted to the ICU, where he was started on levetiracetam 500 milligrams twice a day and treatment for possible CNS infection with ceftriaxone two milligrams twice daily and acyclovir 750 milligrams four times a day. He was also placed on midazolam infusion for sedation. CT scan of the brain on admission was unremarkable and cerebrospinal fluid analysis ruled out any CNS infection. The patient was weaned off ventilator support without any adverse events after 24 hours and antibiotics were discontinued. History obtained after extubation revealed a prior diagnosis of seizure disorder five years ago. The patient was prescribed treatment at the time, but it was discontinued after two years. The patient did not recall the name of the anti-seizure agent prescribed to him previously. Upon admission, initial laboratory investigations, including prolactin, were unremarkable except creatine kinase (CK) levels at 392 IU/L (reference range 22–269 IU/L), which was consistent with seizures. On day three of admission, the patient remained conscious, alert, and oriented with stable vital parameters. Systemic physical examination was unremarkable, and all lab findings, including electrolytes and venous blood gases were within the acceptable limits except for CK that had a sharp 10-fold rise for no apparent reason. This rise in CK led to a consequent deterioration in renal function that was previously normal. There was no clinical or laboratory evidence to suggest sepsis and urine output remained steady at 30 ml/hour. The patient had no symptoms of rhabdomyolysis, such as myalgia, weakness, or muscle cramps. Since the most sensitive indicator of rhabdomyolysis is elevated CK levels [11], the patient was treated for rhabdomyolysis with aggressive intravenous fluids without any response regarding the CK or the creatinine, both of continued to deteriorate and precipitated pulmonary oedema necessitating one session of hemodialysis. As the only drug the patient was on at that time was levetiracetam, iatrogenic cause was suspected leading to this being replaced by phenytoin on day six after admission. Immediately after stopping levetiracetam, there was a decrease in CK levels and within three days of stopping the medication, there was almost halving of the CK with a subsequent, albeit slow decrease in the creatinine level. The patient's renal function improved in tandem with the decreasing CK levels upon discontinuation of levetiracetam as shown in Figures 1 and 2. Follow-up of the patient two weeks after discharge showed normalized levels of creatinine and CK with no further seizures reported.

## 3. Discussion

Levetiracetam is a relatively new anti-seizure. It was approved for clinical use by the FDA in 1999, and in Japan in 2010 as a combination therapy with other anti-seizure drugs for partial onset seizures and secondary generalized seizures for which anti-seizure medications are ineffective. The mechanism of action of this medication remains unknown; however, interactions such as binding to synaptic vesicle protein 2A (SV2A) involved in vesicle exocytosis, inhibition of Ca^2+^ N-type channels, and the neuromodulator action on GABA, 5HT, *α*2-adrenergic, and *μ*-opioidergic pathway have been studied [12]. Large-scale clinical trials on levetiracetam-like the keeper trial, SKATE study and the ASIA SKATE II study reported somnolence (12.8 to 30%), asthma (8.3%), dizziness (7.2–14%), headache (5.9–10.3%), and fatigue as major side effects [13–15].

Rhabdomyolysis occurs as a result of damaged or injured skeletal muscle. Disruption of skeletal muscle causes the release of intracellular constituents, including myoglobin, CK, aldolase, and lactate dehydrogenase, as well as electrolytes, into the bloodstream and extracellular space. Clinical presentation varies from the patient being asymptomatic to severe electrolyte imbalances, acute renal failure, and disseminated intravascular coagulation [11]. Some known causes of rhabdomyolysis include trauma and compression, strenuous exertion, muscle hypoxia, hypo- and hyperthermia, seizures, infection, electrolyte imbalances, autoimmune disorders such as polymyositis and dermatomyositis, certain genetic defects, and certain drugs [16]. There are other anti-seizure medications reported to cause rhabdomyolysis such as phenytoin, valproic acid, gabapentin and lamotrigine [17].

The cause of rhabdomyolysis induced by levetiracetam remains unknown, but some clues have been linked with the medication's ability to potentiate cholinergic function, which is thought to lead to increased neuromuscular transmission, and thus results in muscles being exposed to levetiracetam to be in an energy depleted state rendering them more prone to injury [18, 19]. The mild safety profile, yet high efficacy in treating partial onset seizures for which other antiseizures have failed, has led to levetiracetam being widely used. Despite this extensive use of the drug, rhabdomyolysis as a side effect remains uncommon and possibly underreported adverse event until recently, where a recent pharmacovigilance study showed levetiracetam as having the highest association with rhabdomyolysis among newer-generation antiseizure medications [10].

Acute kidney injury (AKI) associated with levetiracetam-induced rhabdomyolysis has variable incidence as previously reported [20–24]. However, to the best of our knowledge, this is the first case reported to have AKI severe enough to require hemodialysis. This emphasizes the importance of early recognition of this adverse effect as the severity can lead to significant morbidity, and possibly mortality.

In the case of our patient, causality cannot be established without reintroduction of the medication, which is unethical. Although elevation of CK levels can occur in patients after seizures, and the time frame for such elevation can be similar to that seen in our case [25], the absence of a documented generalized tonic clonic seizure, presence of other similar case reports [1–9], and the fact that the patient was on a single medication and a drop in CK levels after stopping the medication helps aid the linkage. It is also worth noting that the serum prolactin level was normal on admission, as this helps aid us in considering alternate causes of the elevated CK than seizures as elevated prolactin has been associated with generalized tonic clonic seizures [26]. That being said, the time interval to CK elevation after the introduction of levetiracetam seen in our patient at day six was in conjunction with other reports occurring between days 3–8, with only one study reporting peak elevation at day 15 [16]. Peak CK level in our patient was at 62299 IU/L, which was significantly higher than other reported cases in which the maximum was 49539 IU/L at day five [27]. Finally, the rapid decrease in CK level following withdrawal of the medication with subsequent normalization of CK level was seen. A similar downtrend of CK levels immediately after discontinuation of levetiracetam has been noted in other studies as well which also helps establish causality.

In conclusion, levetiracetam is a widely used antiseizure medication with multiple known, but not so severe, adverse effects. Rhabdomyolysis associated with the use of this medication is a complication reported but not yet recognized widely. The severity of this complication ranges from being asymptomatic to significant morbidity including AKI and the need for hemodialysis as observed in our case. Therefore, routine monitoring of CK levels during the early phase of treatment, mainly during days one to eight of treatment can lead to an early recognition and management of this complication.

## Data Availability

The clinical data of this patient are available within the article.
